# Navigating Confidentiality Dilemmas in Student Support: An Institutional Ethnography Informed Study

**DOI:** 10.5334/pme.1151

**Published:** 2024-03-12

**Authors:** Emmanuel Tan, Grainne P. Kearney, Jennifer Cleland, Erik Driessen, Janneke Frambach

**Affiliations:** 1 Lee Kong Chian School of Medicine, Nanyang Technological University, Singapore; 2 Maastricht University, Maastricht, The Netherlands; 3 Centre for Medical Education, Queen’s University Belfast, Belfast, United Kingdom; 4 Medical Education Research and Scholarship Unit, Lee Kong Chian School of Medicine, Nanyang Technological University, Singapore; 5 Department of Educational Development and Research, School of Health Professions Education (SHE), Faculty of Health, Medicine and Life Sciences, Maastricht University, Maastricht, The Netherlands; 6 Department of Educational Development and Research, School of Health Professions Education (SHE), Maastricht University, Maastricht, The Netherlands

## Abstract

**Introduction:**

School-level student support programmes provide students with pastoral care and support for academic, wellbeing and other issues often via a personal tutor (PT). PT work is a balancing act between respecting the confidential information divulged by students and doing what is expected in terms of accountability and duty of care. We aimed to explore how tutors manage this tension, with the aim of advancing understanding of student support programmes.

**Methods:**

This qualitative study was informed by an Institutional Ethnography approach. We conducted 11 semi-structured interviews with PTs from one medical school in Singapore. We considered how they worked in relation to relevant national and institutional-level policy documents and reporting guidelines. Data collection and analysis were iterative.

**Results:**

We crafted two composite accounts to illustrate the dilemmas faced by PTs. The first depicts a PT who supports student confidentiality in the same way as doctor-patient confidentiality. The second account is a PT who adopted a more mentoring approach. Both tutors faced confidentiality challenges, using different strategies to “work around” and balance tensions between accountability and maintaining trust. PTs were torn between school and student expectations.

**Discussion:**

Fostering trust in the tutor-student relationship is a priority for tutors but tensions between confidentiality, accountability and governance sometimes make it difficult for tutors to reconcile with doing what they think is best for the student. A more nuanced understanding of the concept of confidentiality may help support PTs and ultimately students.

## Introduction

Student support is increasingly necessary given the rising prevalence of depression, stress, and anxiety amongst medical students [1]. As part of their duty of care [2], many medical schools have developed and implemented support mechanisms ranging from targeted interventions focusing on specific wellbeing concerns to school-wide wellbeing programmes aimed at fostering holistic wellbeing [3,4].

While support systems are welcome, our experience is that there are often tensions between the institutional expectations for reporting and record keeping and the need to build trust and relationships between those tasked with student support and the students they support [5,6]. For example, institutional protocols on documentation can create a sense of surveillance [5]. Students may feel more hesitant to seek help and disclose their wellbeing struggles if they think their conversations may potentially be shared with others, with a possible negative impact on their future medical careers [7].

We do not know how those staff offering support navigate this dilemma; how they balance reporting and confidentiality. This is important to understand as breaking confidentiality is likely to adversely impact the trust between student and member of staff, and thus jeopardise the staff-student relationship and ultimately the success of the student support programme [8,9,10]. Staff may also worry about managing confidentiality appropriately because of potential medico-legal claims [11]. This dilemma between reporting and confidentiality was highlighted as a recurring tension in the staff-student relationship in a recent review of school-wide wellbeing programmes [12].

To examine this tension further, we adopted institutional ethnography (IE) as an organising framework to explore the relationship between the institutional protocols on wellbeing and the everyday experiences of those who provide support. The IE approach focuses on the practical dimensions of how the work of supporting students happens, as well as making visible the complex and often hidden institutional and discursive practices which may influence the choices that staff, in this case, personal tutors (PTs: an assigned faculty member to provide on-going support for pastoral care and support for academic, wellbeing and other issues) [13,14,15], made in discharging their duty of care. The PT’s perspectives or ‘standpoint’ in the IE context further allows us to further examine the broader organisation of student support and power dynamics involved.

Our aim in this paper is to provide a comprehensive understanding of the tensions and complex work involved in maintaining confidentiality while building trust in the area of student support. We explored the PT-student relationships, their interactions, and record-keeping activities in relation to student support. We ask this question: what is the work of PTs in navigating the delicate balancing between respecting and disclosing confidentiality?

## Methods

We conducted a qualitative study, applying the approach of institutional ethnography (IE) in an informed way [16]. Congruent with our research interest, IE focuses on people’s routine work and work processes, set in an institutional order. As a critical sociological approach, it emphasises the role of texts and discourse as a means to coordinate work processes, and how institutional processes and dynamics come to shape and influence the individuals’ experiences within an institution [17,18]. An IE informed approach applies key IE concepts, such as work, text, and discourse but prioritises practicality and utility in data collection and analysis. Our focus was to unpack and explicate the relationship between personal tutors’ ‘everyday experiences’ of student support, as well as the ‘institutional rules’ governing these processes, such as the policies, guidelines, and procedures related to student wellbeing and their support provided by their personal tutors.

### Context

This study was conducted at Lee Kong Chian School of Medicine (LKCMedicine), Nanyang Technological University (NTU), Singapore. LKCMedicine is a joint venture between Imperial College London and NTU. This partnership which reflects the history and present of Singapore whose culture and heritage are influenced by its historical past of British colonialism but its present focus is emphasising its Asian-ness [19].

Student wellbeing and support in the undergraduate medical degree programme at LKCMedicine is delivered through a ‘House System’, where students are assigned to one of five nominal ‘houses’ when they first matriculate. The House System assigns each student a PT, whose main role is to mentor, guide and support the students’ overall wellbeing via regular one-on-one discussions over the five years of the medical degree programme. PTs are largely comprised of general practitioners in private practice (approximately half of the PTs), other doctors from Singapore’s public healthcare institutions and LKCMedicine faculty members (mostly biomedical scientists). Each PT works with about 20 students across the different year groups. The PT is not involved in academic support. However, PTs may be involved in other aspects of teaching within the medical school curriculum.

Confidentiality is typically a key part of the PT role and conversations with students are usually considered confidential. However, PTs are required to maintain an electronic record of their meetings with the students, for record keeping and for the school to ensure that the mandatory one-on-one meetings have taken place. The records are accessible only to authorised staff involved in supporting the students, except when there are regulatory requirements or when there is a risk to self or others. All PTs receive regular training and updates on policy and skills on supporting students, including guidance on what records to keep and what to do with disclosures.

### Participants

We sent invitation emails to all LKCMedicine PTs (about 45 in total). We explained the purpose of the study, what it involved, and that participation was voluntary. We then followed up with those who expressed an interest in participating by arranging a convenient time and place for an individual interview. We took care to include tutors from all five houses, different genders and professional backgrounds, with the intent of achieving maximal sampling variation (Sargeant, 2012).

### Data collection

Drawing on the literature and our previous work on this topic [12], we designed an interview guide (see Supplementary File 1: Appendix) to elicit PT views of their role, what they do and how they carry out their routine one-on-one discussions with students. At the beginning of each interview, we explained the purpose of the study and reminded participants that they could skip any questions and stop the interview at any time if they felt uncomfortable. The follow-up interviews were conducted to follow up themes in the original interviews, and probe deeper into why and how the tutors navigated confidentiality issues. Again, we tried for diversity, purposively following up medical and non-medical tutors.

In line with the IE methodology [20] and because of the growing awareness of the role of documents in health profession education [21], we collected texts mentioned by participants in interviews and those we felt might provide useful context and background information, both to consider during the interviews and to help us make sense of the interview data. These included ‘boss’ texts, such as the Singapore Medical Council’s ethical code and guidelines, which are documents positioned at the top of the hierarchy in relation to other texts [22,23]. It also provided the context from which ground levels texts, such as various House System guides and local school policies, were derived and used to coordinate the work of PTs and students within the institution (see Supplementary File 2: Appendix).

### Data analysis

The interviews were audio-recorded with participants’ permission, transcribed verbatim and anonymised. In line with the IE approach, both the interview data and texts were analysed inductively, focusing on how the experiences of PTs are influenced by the roles of institutional practices, power dynamics and texts – how the tutors put the texts into action in their work. Data analysis was led by ET and GK. After several rounds of iterative reviews of the data, a thread of inquiry was developed, with consensus amongst all team members to focus on confidentiality. The team worked collaboratively, via regular Zoom meetings and email conversations, to identify and refine their understanding of the work confidentiality involved for the tutors, how they used texts in this work and what impact navigating the confidentiality challenges had on tutors themselves.

The refined analysis was used to build composite accounts for the reporting of our results. A composite account provides a rich description of the dilemmas and complexities confronted by the personal tutors with regards to confidentiality issues, whilst protecting their identities [24].

### Reflexivity

We acknowledge our subjectivity and the potential influence of our backgrounds, experiences and positionality on the interpretation and analysis of the findings [25]. JC and ET hold local leadership positions and are known to participants. The PTs reported to JC and ET, who are also responsible for reviewing performance and renewing appointments. We addressed potential power issues by reassuring participants on the anonymity and confidentiality of their identities. GK, JF and ED brought outsider perspectives, providing fresh thoughts and insights on institutional processes and norms. We regularly examined our assumptions and preconceptions throughout the project via virtual meetings and an audit trail was maintained to document the developing interpretation and decision making [26]. Our own experiences with navigating confidentiality in mentoring and tutoring students contributed to shaping the problem statement and rationale for the study, and shaped our interpretations by regularly considering how participants navigated the tension between trust and accountability.

### Ethical approval

The study protocol (IRB-2022-259) was approved by the Institutional Review Board of Nanyang Technological University.

## Results

We conducted 11 semi-structured interviews (average length of interview was 40 minutes). ET conducted a first round of seven interviews in July 2022 and follow-up interviews with four of the original interviewees in February 2023. Reflecting the composition of the personal tutor group, we interviewed three General Practitioners (GP) and four basic scientists; four who identified as male and three as female. Of these seven interviewees, two GPs and two scientists also took part in follow up interviews.

We report our results in the form of two composite accounts. These accounts were constructed by the research team, drawn from the entire dataset of interviews, texts and policies. Each account is a carefully constructed narration comprising of direct quotations from a mix of interview transcripts to add depth and richness as well as highlighting specific perspectives. For example, the two composite accounts illustrate contrasting approaches to managing the tension of navigating confidentiality. The accounts are summarised as follows:

1. Audrey – *We should treat our students the same way we treat our patients when it comes to matters regarding confidentiality*.
2. Ramesh – *I’m a mentor, not a doctor to the students. Doctor-patient confidentiality is inappropriate as a guiding principle for how I support my students*.

Weaving direct quotes from participants into the account (as italics), the composite accounts revolve around three key themes: 1) the differing views on the confidentiality standards to apply: that of doctor-patient relationship or as personal tutors in an academic setting; 2) the strategies and workarounds the tutors employed to navigate challenges; and finally, 3) the central role of trust in this balancing act.

### The story of Audrey

Audrey is a GP with many years of experiences as a house tutor supporting medical students. In the course of her PT work, Audrey has faced several challenges in navigating the delicate balancing act between respecting the confidential information that the students confided in her and deciding whether she should break confidence and inform the school. These included students divulging psychiatric conditions and mental health struggles. She related how students were sometimes very clear that they did not want to tell anyone in the school about their challenges.

She navigated these challenges by exercising the same standards that she practices with patients as a GP in the clinical setting. In fact, she applied the concept of confidentiality in a way that is similar to the approach she uses with patients - as something that is sacrosanct and must be managed with great care.

*“… how we treat our patients, the doctor-patient relationship, I also use it in my tutor-student relationship. Like if they share with me something sensitive, I ask their permission for me to share with other people or not.” (P12)*

As a result, Audrey often felt conflicted on whether to record the details of the discussion or sensitive information about her students in the institution’s official records.

*“And also for the form, because it goes into the file, I don’t really put anything that’s too detailed or personal in it. So for me usually, erm, the students and I will fill the form out together, now that it’s become a routine thing. Erm we’ll just fill in the topics that we spoke about and anything that we need to follow up on …” (P11)*

The text below illustrates the wordings of the House System policy’s requirement on record keeping that was referred to by Audrey.

*“It is compulsory to submit the confirmation of meeting records after meetings with students, especially one-on-one meetings where concerns and issues are raised that may bear relevance on the welfare of the student …” (House Tutor Handbook, p10)*

Nevertheless, she acknowledged there might be some differences between the doctor-patient and personal tutor-tutee relationships. She explained:

*“(The patient) picked me as his doctor and long term he’s sticking with me, and then this also [fits] under the professional ethics doctors code ah, as a patient, is classified as a doctor-patient relationship, I cannot break that confidentiality. No circumstances (will I break it). Except if the patient wants to commit suicide or something then I have to call the ambulance and send him to (a) hospital …” (P02)*

In order to protect *“the secrets which are confided in me”* (Singapore Medical Council, 2016, p. 10), Audrey carefully adopted ‘workarounds’ to navigate confidentiality challenges. She recalled one incident where her workaround was to first seek the student’s permission to consult a fellow personal tutor, who also practiced as a psychiatrist, for advice. She felt that by doing so she could preserve the confidentiality of her student whilst providing appropriate care and support.

*“… in our house tutor side also we have a psychiatrist, Dr. [x]. So I will ask the student uh, you know, we have a psychiatrist who is uh, expert in, in this field, would you also want me to ask him how to do uh, you know, I mean for your case. Of course, I’m, we are doctors, … I also treat patient with depression uh, you know, anxiety and all those with medication you know, in my clinic. But at the same time, we want to give the best care to the student so I ask for permission.” (P12)*

The doctor-patient standards to which Audrey referred are the Singapore Medical Council’s Ethical Code and Ethical Guidelines (ECEG) (2016 edition) (Singapore Medical Council, 2016). These standards outline the obligations of medical practitioners with regards to respecting patient autonomy and maintaining strict confidentiality, as well as specifying the circumstances under which disclosure of confidential patient information may be permitted. In the last sentence of the quotation, Audrey used the word ‘… to give the best care …’. The term ‘care’ is also a term that is referred to numerous times throughout ECEG in relation to ‘clinical care’, ‘duty of care’, and others. In short, her approach towards navigating complex ethical dilemmas and confidentiality issues around student support was closely aligned to the principles and concepts governing the medical profession.

Audrey also believed that trust is a fundamental aspect of the personal tutoring relationship. She feels her students would expect her to uphold the same level of confidentiality as her patients would expect of her as a doctor. She believed that maintaining this level of confidentiality was beneficial for the students. Audrey acknowledged that the consequences of mismanaging confidentiality can be grave, both for the student and for the relationship between the student and the tutor. In all, trust underpins all her work as a PT.

*“A lot of the time I feel that the, the things that you know, they, they need the trust. They need trust you know, in the tutor. And they also need to know that you are willing to help them so this two things, if no trust, you don’t develop trust and you don’t develop the fact that you are willing to help them, most of the time they, they will not share … so in the end I realised that, in order to achieve that, you have to really sit down and talk to them and know them intimately in terms of the fact that the point that they can feel that they trust you too. So I will say it’s levels of trust. You must reach that kind of like the, don’t know what, the fifth level maybe, whatever. And that is really a lot of effort and time.” (P02)*

### The story of Ramesh

In contrast to Audrey, Ramesh, a basic scientist firmly believed that in the school context, he is first and foremost a mentor, not a doctor to his students. He was aware that he operates in an environment where many of his colleagues are clinical doctors and may have differing views of how one should navigate confidentiality issues. Despite this, he strongly felt that the doctor-patient relationship is an inappropriate mantra for his work as a PT navigating confidentiality issues: “*here it is not doctor-patient, it’s house tutor and tutee … (if there) are the things to be considered, uh, which should be recorded, then, yes, uh, we have to give, we have to suggest to the student …” (P07)*

Nevertheless, similar to Audrey, Ramesh generally avoided recording any sensitive or confidential information in the electronic system. On one level, Ramesh regarded record keeping as a form of additional administrative responsibility to his PT duties. He felt it was more important to use the time to build relationships with his students.

*“I’ll remind (my students) … can you please, uh, sign the one-on-one form. Then they’ll say okay, so they’ll just sign it off, to just prove that we had that session, ah, otherwise the school will come after us.” (P18)*

But, perhaps more importantly, he had deep concerns around keeping confidential records on file as he was unclear as to how the electronic record would be used and whether the student could see it. He also felt a slight sense of frustration, stemming from the uncertainty on whether reporting is the right course of actions. Consequently, he was very cautious in what he would record as part of the official notes.

*“I don’t know how they’re used but I know like when I worked there, it, it gets recorded. (Does) it goes to the school, wellbeing centre or the education team, and they (students) see the progress? I do know that eventually the documentation will go back into their P (personal) files. That much I know. Beyond that I’m not sure how it goes.” (P07)*

Having said so, Ramesh acknowledged his duty of care towards his students. He understood that in some situations, such as when a student’s safety was at risk, confidentiality must be broken. Where appropriate, he would escalate the concern, by informing the school, the senior house tutor and assistant dean of student wellbeing. However, his preference would be to record students’ concerns and sensitive information in a general way, rather than specifying issues.

*“Okay so, she did have this concern and I thought it was a rather sensitive issue um, and I was in a dilemma whether to record it down. But nonetheless, I thought it’s actually quite a serious issue as well too, and should be documented down. So what I did was to actually uh, put it down on the record but put it in a more uh, a general way you know, but not going into the details. But at least there is some record of you know what are the concerns of a sensitive nature that this tutee is facing.” (P16)*

Reflecting on his PT experiences, he identified trust as the most critical element of his work in navigating confidentiality. Students must trust that tutors want to help. He believed that trust is a fragile thing. It can shatter easily. In fact, the need for trust was what allowed him to explain to his students why confidentiality needed to be broken in extenuating circumstances. He felt that trust is paramount in the personal tutor-student relationship.

*“I think trust is, is extremely important uh, you know in the house system between the tutor and the tutee … more so for a tutor and a tutee because the tutee would look upon you as a role model. And also look upon the tutor as a person to you know seek uh, advice. And not just advice on academic. It can even be advice uh, life advice. You know, advice as pertaining to uh, personal issues. Okay so hence I think it’s extremely important to actually maintain this, this level of trust because um, if the trust were to be broken in any way, the tutee could actually uh, become withdrawn as behaviourally.” (P16)*

## Discussion

The two composite accounts highlight distinct approaches in how PTs navigated confidentiality concerns in student support. Whilst tutors faced similar dilemmas on what to record, how much to record and how their actions could affect the trust in tutor-student relationship, they sought slightly different workarounds and strategies to overcome these challenges while prioritising their students’ best interests.

Institutional policies mandate that the PTs report significant student concerns, such as self-harm. However, the confidentiality dilemmas can be complex and the characters in the two vignettes often faced similar challenges in having to make difficult judgment calls on whether or not to report to the school. Some felt accountable to their students, others to the institution and/or their professional ethical code and guidelines. Trust was highlighted as paramount by all the personal tutors regardless of their approach in navigating confidentiality.

How personal tutors used and related to the concept ‘confidentiality’ largely aligned with the notion that confidentiality is a ‘god term’ [27,28,29] and an institutional discourse [17,30] in student support. To elaborate, confidentiality is a widely accepted, unquestioned principle that is cherished for its role in promoting trust and building relationships, and a principle to be fervently preserved except in exceptional circumstances such as when there is a potential harm to the student or others, or if it is required by law to disclose the information. Confidentiality can also be described as an institutional discourse that reflects the PTs respecting secrets and maintaining trust, in an unquestionable way that it is (considered to be) in the students’ best interests. We suggest that PTs and individuals engaged in student support must critically examine their understanding of confidentiality and be aware that their professional roles and obligations may at times conflict with institutional policies and guidelines.

Culture is likely to play an important role here. Our study was carried out in Singapore, a country where people generally place high trust in institutions and authority figures, and there is a strong emphasis on accountability [31]. Yet, we also observed individualism and personal autonomy, cultural norms which have traditionally been ascribed to western cultures [32], in tutors’ reluctance to adhere strictly to the institution disclosure obligation. This phenomenon reflects Singapore’s heritage, as mentioned earlier, one that encompasses both western and Asian cultural norms as the nation searches for its own postcolonial identity. Viewed through this lens, the PTs’ deliberations and actions on student confidentiality dilemmas in some ways reflect the larger, national cultural context and dynamics at play. The complexity of these cultural factors raises questions about the dynamic and complex role of culture in respect of professional obligations and accountability in different societies [33]. More cross-cultural comparative research on this topic would be valuable [32].

Irrespective of culture, the overlapping layers of accountabilities inherent in student support systems are not always reconcilable. Who should the PT be accountable to, and for what purposes? How does their sense of accountability promote or hinder their work in student support, and how does it shift depending on the specific needs of the student? By exploring the complexities of these confidentiality dilemmas in student support we can also shed light on other related areas of medical education whereby confidentiality is a key concern (e.g. professionalism [34], assessment [35], patient safety [36], use of electronic files and portfolios [35], learner analytics [37], and many other related areas [38,39,40,41].

Our findings also highlight issues with the use of electronic records in student support. Personal tutors did not always keep electronic records as required by local policy. This was not due to technical barriers. Rather tutors were concerned about safeguarding student confidentiality and maintaining trust, and the potential use of reflections and e-portfolios as legal evidence, as per the UK’s Bawa-Garba case [42]. The impact of high-profile cases on how tutors navigate tensions (and students’ willingness to come forward to seek help) is an important area for future research.

Given this, it would be timely to consider the concept of governmentality in student support. Institutional Ethnography (IE) focuses on structures of power. Drawing on Foucault’s notions of power and the panopticon [43] the work of PTs (regular meetings, recording information) could be interpreted as pervasive monitoring and surveillance of students, and the hierarchical nature of the power relationship between school and students. Students resist this surveillance with tactics such as offering limited information on their wellbeing or delaying help-seeking behaviours. What was surprising in our study was that the tutors, while tasked with surveillance, simultaneously contributed to students’ resistance against the panopticon by helping them evade surveillance (by not keeping records as required by the School). Interestingly, the panopticon is omnipresent [44]. It was not just the students who were aware that they were being observed and evaluated. The personal tutors were equally aware of being monitored by the school in respect of doing what was expected in terms of record keeping, mandatory meetings, etc (as well as being judged by the students on their willingness to maintain confidentiality and build trust). Who was really under surveillance: was it the students or was it the personal tutors? How does this surveillance impact the wellbeing of personal tutors? Perhaps related to this, we sensed an underlying tone of emotional burden carried by the personal tutors when they discussed the need to employ workarounds and navigate institutional record-keeping requirements. Our study questions did not allow us to explore the potential personal impact of confidentiality dilemmas on personal tutors in any depth, but the wider literature suggests this is a phenomenon [45,46,47,48]. We raise these as areas for future research, to advance our knowledge on tutor wellbeing and the difficult issues of confidentiality.

### Implications for policy and practice

Most student support programmes have a guidance policy that guides how they should manage student confidentiality issues [49]. However, our findings suggest that these guidelines may be secondary to PT experience. This leads to challenges and tensions for PTs in respect of adhering to school policies but doing what they think is best for the student. This tension may be helped by schools shifting from considering confidentiality as an unquestionable concept to instead clearly defining and articulating their position on confidentiality in the student-tutor relationship. Schools should engage PTs in critical discussions and open dialogue to challenge prevailing discourses surrounding confidentiality – for example, whether it is in the student’s best interests for PTs who are medical doctors to interact with them in the same way as their patients.

### Strengths and Limitations of this study

Our study is set in one medical school in one context, with one approach to student support delivered mainly by GPs and biomedical scientists. Our findings thus may not be fully transferable to other systems, structures, contexts or cultures. Further research is needed to gain more understanding of the dilemma between confidentiality, trust and governance in different contexts.

Our data hints that culture may be an important influence on professional obligation and tutor emotional burden: this requires further exploration in future studies. This study is one of the first studies to look at the issues of confidentiality in the student-tutor relationship from the PT’s perspective, and so extends the literature on this topic (previous studies have focused on the organisational or students’ perspectives [50,51]). Our use of IE offers a lens to understand how things happened (e.g. the influence of institutional rules on practices) rather than just what happened or was experienced.

As discussed earlier, two of the research team had positions of power in the institution and system. However, we did not sense that tutors felt obliged to participate, or to give socially acceptable responses (indeed they were quite open at pointing out the shortcomings of the House system), and the wider research team had a good balance of “insider” and “outsider” positionality.

## Conclusion

This study uses an IE informed approach to examine the work of personal tutors in navigating confidentiality issues in role of supporting students. Fostering trust in the tutor-student relationship is a priority for tutors but tensions between confidentiality, accountability and governance sometimes make it difficult for tutors to reconcile with doing what they think is best for the student. We suggest that a more nuanced understanding of the concept of confidentiality would help support PTs and ultimately students.

## Data Accessibility Statement

The datasets are available upon reasonable request by contacting the corresponding author.

## Additional Files

The additional files for this article can be found as follows:

10.5334/pme.1151.s1 Supplementary File 1. Appendix.

10.5334/pme.1151.s2 Supplementary File 2. Appendix.
