# Supplementary material for: Navigating Confidentiality Dilemmas in Student Support: An Institutional Ethnography Informed Study
Source: Perspect Med Educ. 2024 Mar 12;13(1):182–91. doi: 10.5334/pme.1151 (PMC10941695; doi:10.5334/pme.1151)
Supplement: Supplementary File 1. — Appendix. [file pme-13-1-1151-s1.pdf]

Supplementary File 1: Appendix

Semi-structured interview guide

1. What is your role in the House System?
2. How has your experience of the House System been so far?
3. How clearly do the House System's policies and local documents spell out what is required for students and staff?
4. What are the main strengths and weaknesses of the House System?
5. What are your views regarding the practicalities of the House System?

Examples of themes we explored in the follow-up interviews

1. Tell me about a time when you were unsure whether or not to record your discussion with your tutee?
2. When this happens, what do you normally do? Are you aware of any guidance?
3. How do you feel managing these situations? Who do you feel accountable to? What makes you feel this way?
4. On doctor-patient / tutor-student relationship,
    - Having spoken to a number of tutors, it seems like we apply the concept of confidentiality in our support relationships with students the way it might apply in a doctor-patient relationship (in a sacrosanct way that may have grave consequences if mismanaged). I wonder what are your reflections on this?
    - What are your views about confidentiality in the doctor-patient relationship? How do you feel it compares with the tutor-student relationship?
