# Supplementary material for: Navigating Confidentiality Dilemmas in Student Support: An Institutional Ethnography Informed Study
Source: Perspect Med Educ. 2024 Mar 12;13(1):182–91. doi: 10.5334/pme.1151 (PMC10941695; doi:10.5334/pme.1151)
Supplement: Supplementary File 2. — Appendix. [file pme-13-1-1151-s2.pdf]

Supplementary File 2: Appendix

"Boss" Texts

1. Singapore Medical Council (SMC)'s Ethical Code and Ethical Guidelines (ECEG) (2016 edition)
2. SMC Handbook on Medical Ethics (2016 edition)
3. SMC Physician Pledge (SMC ECEG, p10)

Local policy and guidance documents

1. House System Guide for Students
2. House Tutor Guide
3. Concern Form for Medical Students
4. Mitigating Circumstances Form
5. Leave of Absence Application Form
6. Interruption of Studies Application Form
